# Trait positive empathy and professional identity among nursing undergraduates: the mediating role of career resilience

**DOI:** 10.3389/fpsyg.2026.1894726

**Published:** 2026-08-10

**Authors:** Jie Liu, Shuxian Cheng, Yaqi Wu, Lisha Zhao, Yuanmei Qin, Xiaolei Jing

**Affiliations:** 1 School of Nursing (Nursing School of Smart Healthcare Industry), Henan University of Chinese Medicine, Zhengzhou, Henan, China; 2 The First Affiliated Hospital of Henan University of Chinese Medicine, Zhengzhou, Henan, China

**Keywords:** career resilience, mediation model, nursing undergraduates, positive psychology, professional identity, trait positive empathy

## Abstract

**Background:**

Professional identity is an important indicator of career development and workforce stability among nursing undergraduates. However, the pathways through which positive psychological resources are associated with professional identity remain unclear. This study aimed to examine the mediating role of career resilience in the relationship between trait positive empathy and professional identity among nursing undergraduates.

**Methods:**

A cross-sectional survey was conducted among 617 nursing undergraduates from a university in Henan Province, China. Participants completed the Trait Positive Empathy Scale (TPES), Career Resilience Scale (CRS), and Professional Identity Questionnaire for Nursing Students (PIQN). Pearson correlation analysis was used to examine the relationships among the variables. The mediating role of career resilience was tested using PROCESS Model 4 with 5,000 bootstrap resamples.

**Results:**

The total scores of trait positive empathy, career resilience, and professional identity were 28.07 ± 4.59, 19.86 ± 3.30, and 60.72 ± 10.44, respectively. Trait positive empathy was positively correlated with career resilience (*r* = 0.654, *p* < 0.001) and professional identity (*r* = 0.486, *p* < 0.001). Career resilience was positively correlated with professional identity (*r* = 0.557, *p* < 0.001). Mediation analysis indicated that career resilience partially explained the relationship between trait positive empathy and professional identity. More than half of this association was explained through career resilience, with the indirect association accounting for 56.23% of the total association.

**Conclusion:**

Trait positive empathy was directly associated with professional identity and indirectly associated with professional identity through career resilience among nursing undergraduates. These findings highlight the importance of cultivating positive psychological resources and career resilience to support professional identity development in nursing education.

## Introduction

Professional identity (PI) refers to an individual’s overall cognition and emotional experience regarding the value, professional role, and sense of belonging associated with their occupation, and is considered an important factor associated with career development, professional stability, and future career intentions of nursing undergraduates (Nie et al., 2021). With the increasing demand for healthcare services and the continuous transformation of nursing service models, the stability of the nursing workforce has become a major concern in the field of nursing education (De Vries et al., 2026; Zhong et al., 2024). Previous studies have shown that low levels of professional identity may not only weaken nursing students’ professional engagement and learning motivation, but also increase the risks of burnout, turnover intention, and professional withdrawal, which may be related to the long-term stability of the nursing workforce (Zhong et al., 2024). Undergraduate nursing education represents a critical stage for the formation of professional identity and professional socialization, during which nursing students gradually internalize the values of the nursing profession and develop an understanding of their professional roles through academic learning, clinical exposure, and role adaptation (Fitzgerald and Butcher, 2025). However, with increasing involvement in clinical learning, nursing students are required not only to cope with heavy academic workloads, but also to adapt to complex clinical environments, nurse–patient communication, and professional role transitions. During this process, some nursing students may experience reduced professional enthusiasm, uncertainty regarding career goals, and insufficient professional identity (Pan et al., 2024). Accordingly, identifying factors associated with the development of professional identity has become an important topic in nursing education research, particularly from the perspective of positive psychology. In recent years, increasing attention has been paid to the perspective of positive psychology in nursing education. Studies have suggested that positive psychological resources may be associated with better adaptation to clinical environments and professional development (Malik and Sinha, 2026). Trait positive empathy (TPE) refers to an individual’s ability to perceive, understand, and positively respond to others’ positive emotions (Andreychik and Migliaccio, 2015). Existing evidence has demonstrated that higher levels of trait positive empathy are associated with stronger positive feedback, better interpersonal interaction abilities, and greater perceptions of professional value among nursing students (Telle and Pfister, 2016). Meanwhile, career resilience, as an important adaptive resource for coping with occupational stress and career-related challenges, has also been considered closely associated with the professional development of nursing students.

Although previous studies have separately examined trait positive empathy, career resilience, and professional identity, research investigating the relationships among these three variables remains limited, particularly regarding the mediating role of career resilience in the relationship between trait positive empathy and professional identity. Therefore, this study aimed to investigate the mediating role of career resilience in the relationship between trait positive empathy and professional identity among nursing undergraduates, in order to provide evidence for understanding professional identity and positive psychological qualities among nursing students.

Career resilience (CRS) refers to an individual’s ability to actively adjust and continuously adapt to career development when facing occupational stress, career setbacks, and complex professional environments (Zhang et al., 2023). Previous studies have suggested that career resilience is not only present among registered nurses, but also gradually develops during nursing education (Aryuwat et al., 2023). During clinical practice, nursing students are continuously exposed to challenges such as academic pressure, clinical adaptation, nurse–patient communication, and professional role transition. Prolonged exposure to high-stress environments may be associated with emotional exhaustion, professional withdrawal, and reduced professional identity (Zhao et al., 2024). Studies have shown that nursing students with higher levels of career resilience generally demonstrate better career adaptability, psychological wellbeing, and professional stability (Wu et al., 2022). Therefore, career resilience is considered an important psychological resource associated with professional adaptation and the development of professional identity among nursing students.

**Figure 1:**
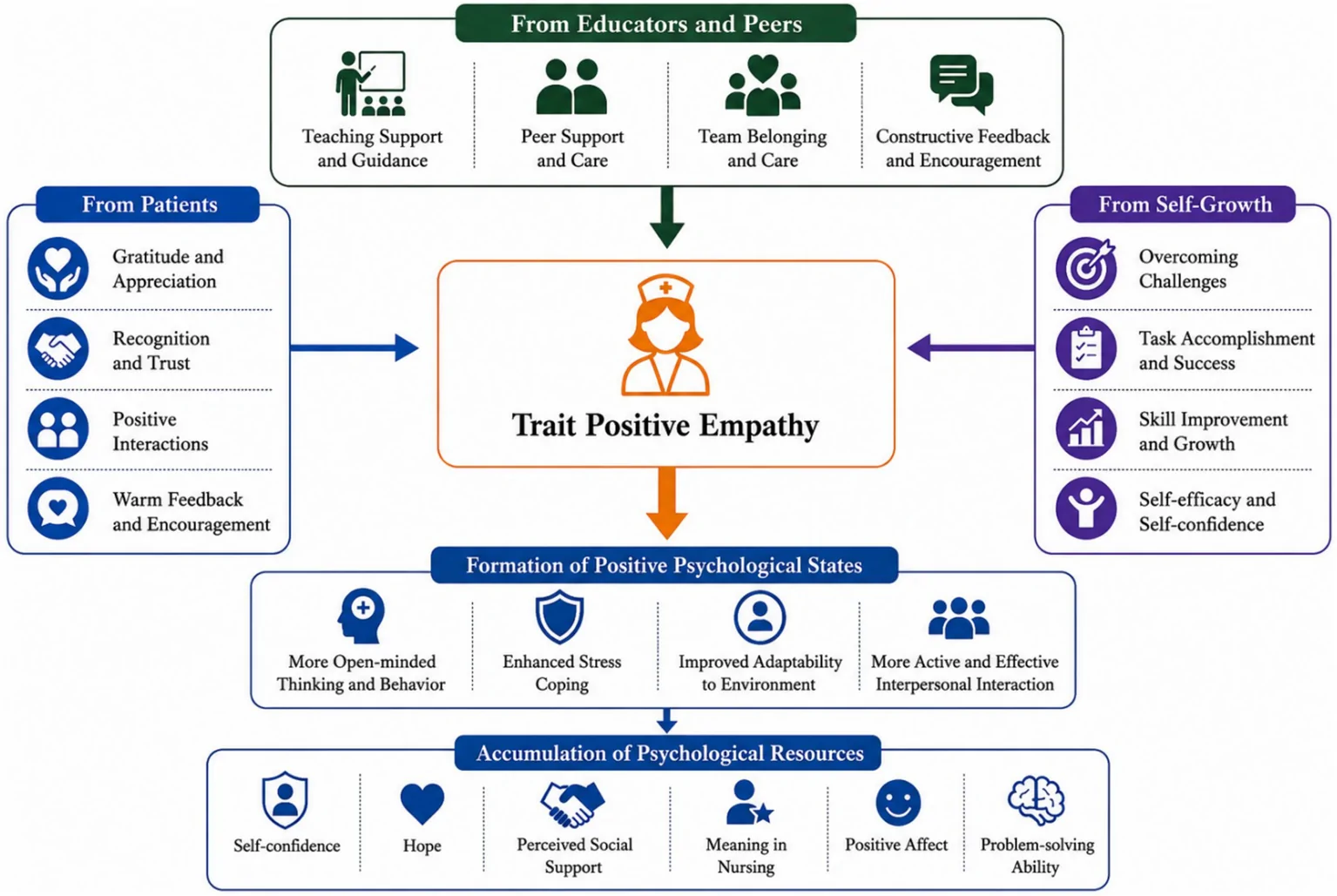
Positive psychological correlates of trait positive empathy among nursing undergraduates.

The development of career resilience is associated with multiple factors, including individual psychological traits, social support, and professional environments. Previous studies have demonstrated that higher levels of trait positive empathy are associated with greater sensitivity to positive feedback from others and more open and proactive engagement in social interaction and interpersonal communication (Yi et al., 2025). For nursing undergraduates, this positive tendency toward interpersonal perception and interaction may be related to better perception of positive information conveyed by patients, educators, and peers during clinical learning, which may be associated with active participation in nurse–patient communication, teamwork, and clinical practice. Fredrickson’s Broaden-and-Build Theory, proposed in 1998, suggests that positive psychological traits and positive emotions may broaden individuals’ momentary cognition and behavioral repertoires, thereby being associated with the accumulation of psychological resources such as adaptability, stress-coping ability, and social support (Roth et al., 2024). Evidence has indicated that positive psychological states are associated with more proactive and optimistic responses to external environments and with psychological stability under stressful conditions (Wu et al., 2020). Compared with negative emotions, which are more likely to trigger avoidance, tension, or defensive reactions, positive psychological states are more likely to encourage individuals to communicate actively with others and solve problems proactively (Ewert et al., 2024). During nursing education, nursing students are required to continuously cope with practical challenges such as academic pressure, role transition, adaptation to clinical environments, and nurse–patient communication. Nursing students with higher levels of trait positive empathy are more likely to perceive positive feedback from nurse–patient interactions, educator guidance, peer support, and personal growth experiences (Figure 1). For example, in nurse–patient interactions, patients’ gratitude, trust, and positive feedback may be related to nursing students’ sense of professional meaning. In learning and team environments, educator guidance, peer support, and team care may help nursing students develop a stronger sense of belonging and support. Moreover, experiences such as overcoming difficulties, accomplishing tasks, improving abilities, and achieving personal breakthroughs during individual growth may be associated with higher self-confidence and positive professional perceptions (Su et al., 2021). These positive feedback experiences may be related not only to lower perceived negative effects of clinical stress, but also to more positive psychological states and better stress-coping ability, environmental adaptability, and interpersonal interaction skills, thereby supporting the development of career resilience.

However, positive professional experiences may not necessarily be directly transformed into stable professional identity. During the process of professional socialization, nursing students are still required to cope with complex clinical environments and occupational stress over a prolonged period, in addition to positive feedback. Without sufficient career adaptability, even individuals with high levels of positive empathy may still experience reduced professional confidence and weakened professional identity under persistent stressful conditions (Wu et al., 2024). Therefore, the association between positive psychological resources and stable professional identity may be related to individuals’ internal psychological adaptation. Based on the Broaden-and-Build Theory, it can be inferred that the positive psychological experiences associated with trait positive empathy may be further related to professional identity through career resilience among nursing students.

At present, studies on professional identity among nursing undergraduates have mainly focused on negative factors such as stress, anxiety, and burnout, whereas insufficient attention has been paid to the pathways through which positive psychological resources are associated with professional identity. In particular, the relationships among trait positive empathy, career resilience, and professional identity, as well as their potential pathways, remain insufficiently explored. Therefore, this study further investigated the mediating role of career resilience in the relationship between trait positive empathy and professional identity, with the aim of providing evidence for understanding professional identity and positive psychological qualities among nursing undergraduates. Based on the existing literature and the Broaden-and-Build Theory, the following hypotheses were proposed:

H1: Trait positive empathy is positively associated with professional identity among nursing undergraduates.

H2: Career resilience is positively associated with professional identity among nursing undergraduates.

H3: Trait positive empathy is positively associated with career resilience among nursing undergraduates.

H4: Career resilience plays a mediating role in the relationship between trait positive empathy and professional identity among nursing undergraduates.

## Methods

### Study design and setting

This study adopted a cross-sectional descriptive design and was conducted in accordance with the Strengthening the Reporting of Observational Studies in Epidemiology (STROBE) guidelines (VON Elm et al., 2008). The study was conducted at the School of Nursing of a university in Henan Province, China, where undergraduate nursing students receive systematic theoretical education and staged clinical practice training. This nursing school was selected because it has a complete undergraduate nursing education system, includes students from different academic years, and provides staged clinical practice opportunities, making it suitable for examining the relationships among trait positive empathy, career resilience, and professional identity in nursing undergraduates.

### Sample size and study participants

Nursing undergraduates from the School of Nursing of a university in Henan Province were recruited using a convenience sampling method. Eligible participants were undergraduate nursing students enrolled during the 2024–2025 academic year. Participants were recruited through class-based announcements with the assistance of nursing faculty and class advisors. Before questionnaire distribution, the researchers introduced the study purpose, inclusion criteria, questionnaire content, estimated completion time, and confidentiality measures to eligible students. Students who met the inclusion criteria were invited to complete the online questionnaire. Participation was entirely voluntary, and students were informed that they could decline to participate or withdraw from the study at any time without any negative consequences. All participants provided informed consent before completing the questionnaire. During professional learning and clinical practice, nursing students gradually develop an understanding of professional nursing values and roles. Therefore, nursing students across different academic years were considered suitable participants for examining the relationships among trait positive empathy, career resilience, and professional identity.

Participants included first- to fourth-year nursing undergraduates. First-year students mainly focused on theoretical coursework, including education related to professional identity and nursing career development. In addition to professional coursework, second- and third-year students completed a 2-month clinical practicum, during which they began to experience clinical environments, nurse–patient communication, and professional role adaptation. Fourth-year students completed an 8-month clinical internship and participated more extensively in real-world nursing practice. Therefore, nursing students at different academic levels may differ in positive feedback, career resilience, and professional identity, making them meaningful research populations.

The sample size was estimated using the formula *N* = (*Zα*/2σ/*δ*)2 (Althubaiti, 2023). A pilot study involving 30 nursing undergraduates showed that the professional identity score was 59.34 ± 10.26. Accordingly, the sample size was calculated using α = 0.05, the standard deviation obtained from the pilot study, and the allowable error. Considering a potential invalid or missing questionnaire rate of 10–20%, the sample size was further increased. In addition, given that mediation analysis was required in this study, the final sample size was expanded to ensure adequate statistical power.

**Figure 2:**
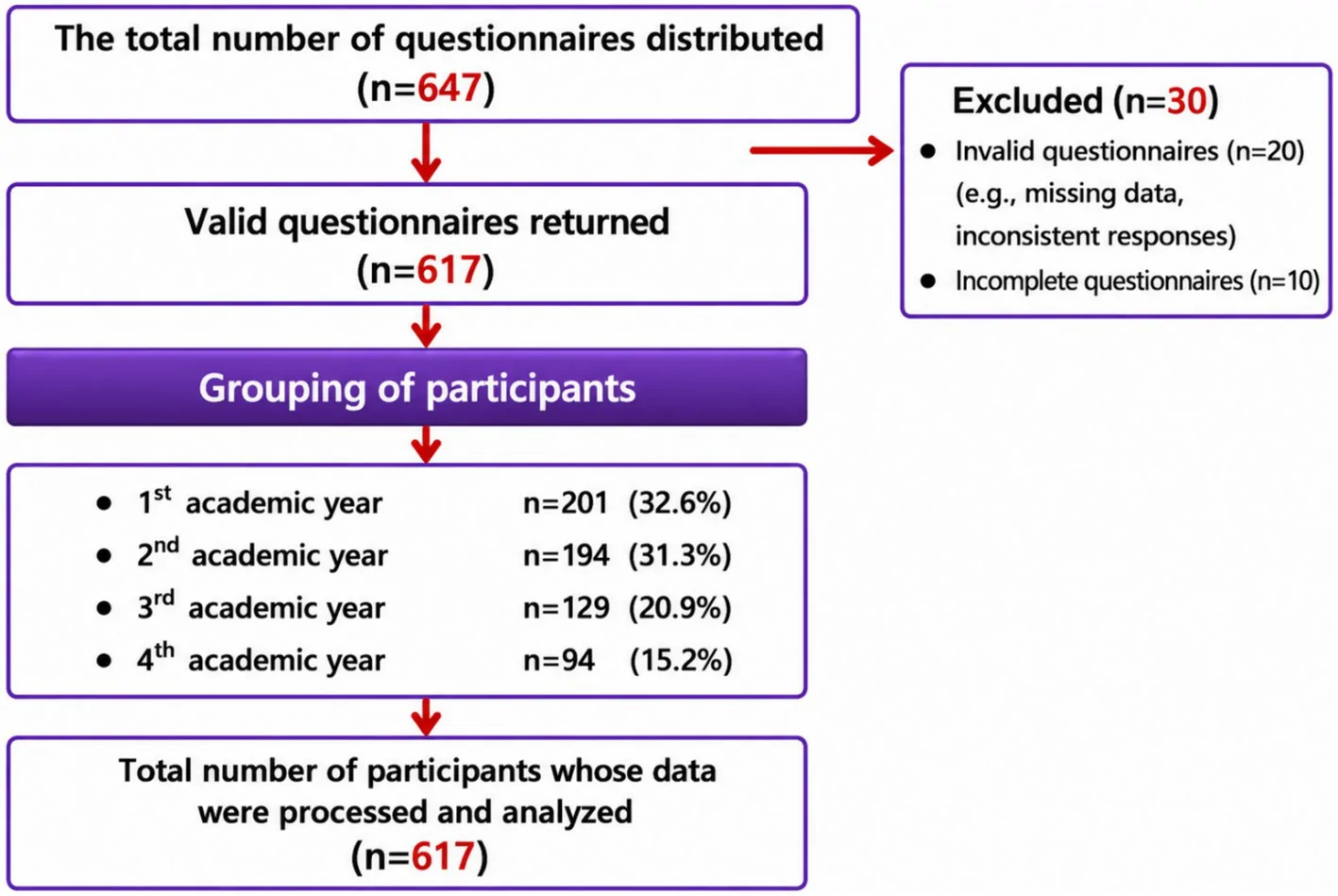
Flowchart of questionnaire screening and participant inclusion among nursing undergraduates.

A total of 647 questionnaires were distributed. After excluding 20 invalid questionnaires and 10 questionnaires with missing data, 617 valid questionnaires were retained, resulting in an effective response rate of 95.36%. Finally, 617 nursing undergraduates were included in the data analysis. To include students with different learning and clinical exposure experiences, nursing undergraduates from different academic years were included, including 201 first-year students (32.6%), 194 second-year students (31.3%), 129 third-year students (20.9%), and 94 fourth-year students (15.2%). The participant selection process is presented in Figure 2.

## Measurements

### General information survey questionnaire

The questionnaire was developed by the researchers based on an extensive review of relevant literature and related studies and collected general demographic information, including grade, gender, place of residence, and whether nursing was chosen as the first-choice major.

### Trait positive empathy scale

The Trait Positive Empathy Scale (TPES), developed by Morelli et al. in 2015 and culturally validated in Chinese by Yue Tong, was used to assess individuals’ levels of trait positive empathy (Morelli et al., 2015). The scale consists of 7 items and uses a 5-point Likert scale ranging from 1 (“strongly disagree”) to 5 (“strongly agree”). Higher total scores indicate higher levels of trait positive empathy. The original Cronbach’s *α* coefficient of the scale was 0.88, and the Cronbach’s α coefficient in this study was 0.939.

### Career resilience

Career resilience was assessed using the Career Resilience Scale (CRS), originally proposed by London in 1993 and later culturally adapted for Chinese populations by Wang Qiaoling (London and Noe, 1997). The scale contains 5 items and adopts a 5-point Likert scale ranging from 1 (“strongly disagree”) to 5 (“strongly agree”). Total scores range from 5 to 25, with higher scores indicating higher levels of career resilience. The original Cronbach’s *α* coefficient of the scale was 0.933, and the Cronbach’s α coefficient in this study was 0.892.

### Professional identity questionnaire for nursing students

Professional identity was measured using the Professional Identity Questionnaire for Nursing Students (PIQN), developed by Hao Yufang in 2011 (Hao, 2011). The questionnaire consists of 17 items across five dimensions: professional self-concept (6 items), social comparison and self-reflection (3 items), perceived benefits of remaining versus leaving the profession (4 items), autonomy in career choice (2 items), and social persuasion (2 items). The questionnaire uses a 5-point Likert scale ranging from 1 (“strongly disagree”) to 5 (“strongly agree”), with Item 12 scored in reverse. Total scores range from 17 to 85, with higher scores indicating higher levels of professional identity. The original Cronbach’s *α* coefficient of the scale was 0.827, and the Cronbach’s α coefficient in this study was 0.931.

### Study procedures

Before the formal investigation, the researchers refined the study instruments through a literature review and provided standardized instructions to participants regarding the study purpose, questionnaire requirements, and precautions. To improve the comprehensibility and quality of the questionnaire, a pilot study involving 30 nursing undergraduates was conducted prior to the formal survey, and minor modifications were made based on the pilot results. The pilot results indicated that the scale items were clearly expressed and easy to understand; therefore, no further modifications were made to the instruments. During the investigation, questionnaires were uniformly distributed and collected by the researchers, who also informed participants that the study followed the principles of informed consent, voluntary participation, anonymity, and confidentiality. Participants were free to decide whether to participate and could withdraw from the study at any time without penalty. All questionnaires were completed independently by the participants, while researchers provided on-site guidance and answered questions in a timely manner to support the quality of questionnaire completion.

### Data collection

Data collection was conducted from September 2025 to January 2026. Before the survey, the researchers explained the study purpose, survey content, and questionnaire requirements to nursing undergraduates and emphasized that the study adhered to the principles of informed consent, voluntary participation, anonymity, and confidentiality, and that all data would be used solely for research purposes. After obtaining informed consent, the survey was conducted using an online questionnaire. The questionnaires were uniformly distributed by the researchers and completed and collected collectively after class sessions. During questionnaire completion, researchers provided on-site guidance and standardized explanations to participants’ questions to ensure data quality. The questionnaire required approximately 10–15 min to complete. After data collection, all returned questionnaires were reviewed by the researchers. Questionnaires with obvious patterned responses, substantial missing data, or incomplete information were excluded, resulting in 617 valid questionnaires for analysis.

### Data analysis

Data were analyzed using SPSS version 27.0. Continuous variables were described using mean ± standard deviation (Mean ± SD), while categorical variables were presented as frequencies (n) and percentages (%). Harman’s single-factor test was conducted to assess common method bias. Pearson correlation analysis was performed to examine the relationships among trait positive empathy, career resilience, and professional identity among nursing undergraduates. The mediating role of career resilience in the relationship between trait positive empathy and professional identity was examined using Model 4 of the PROCESS macro (version 4.1) developed by Hayes for SPSS, with trait positive empathy as the independent variable, professional identity as the dependent variable, and career resilience as the mediating variable. A Bootstrap method with 5,000 resamples was applied, and a 95% confidence interval (95% CI) that did not include zero was considered indicative of a statistically significant indirect association. All statistical tests were two-tailed, with the significance level set at *α* = 0.05.

## Results

Given that all study variables were collected using self-reported questionnaires, Harman’s single-factor test was conducted to assess the potential influence of common method bias. The results showed that four factors with eigenvalues greater than 1 were extracted under the unrotated condition. The first factor explained 43.414% of the total variance, which was below the recommended threshold of 50%, indicating that common method bias was not a serious concern in the present study.

A total of 617 nursing undergraduates were included in this study. Among them, 76.7% were female, 50.2% came from urban areas, and 43.9% reported choosing nursing because of career development prospects. Participants covered all academic years, including 201 first-year students (32.6%), 194 s-year students (31.3%), 129 third-year students (20.9%), and 94 fourth-year students (15.2%). Detailed demographic characteristics are presented in Table 1.

**Table 1:** General characteristics of participants (*N* = 617).

Variables	*n*	%
Grade		
Freshman	201	32.6
Sophomore	194	31.3
Junior	129	20.9
Senior	94	15.2
Residence		
Rural area	307	49.8
Urban area	310	50.2
Gender		
Male	144	23.3
Female	473	76.7
Reasons for choosing nursing major		
Career prospects	271	43.9
Personal interest	91	14.7
Family influence	125	20.3
Major reassignment	60	9.7
Others	70	11.3

The total score of trait positive empathy among nursing undergraduates was 28.07 ± 4.59, with an item mean score of 4.01 ± 0.66, indicating a moderate-to-high level. The total score of career resilience was 19.86 ± 3.30, corresponding to a mean item score of 3.97 ± 0.66. The total score of professional identity was 60.72 ± 10.44, with an item mean score of 3.57 ± 0.61. Among the dimensions of professional identity, “Social Persuasion” and “Social Comparison and Self-Reflection” showed relatively higher scores, whereas “Job Retention versus Leaving Benefits” showed relatively lower scores. Detailed results are presented in Table 2.

**Table 2:** Descriptive statistics of trait positive empathy, career resilience, and professional identity.

Variables	Number of items	Mean score (*x* ± *s*)	Total score
Trait positive empathy	7	4.01 ± 0.66	28.07 ± 4.59
Career resilience	5	3.97 ± 0.66	19.86 ± 3.30
Professional identity	17	3.57 ± 0.61	60.72 ± 10.44
Professional self-concept	6	3.56 ± 0.75	21.34 ± 4.48
Social comparison and self-reflection	3	3.80 ± 0.68	11.40 ± 2.05
Job retention versus leaving benefits	4	3.40 ± 0.75	13.58 ± 2.98
Career choice autonomy	2	3.54 ± 0.71	7.08 ± 1.41
Social persuasion	2	3.82 ± 0.71	7.63 ± 1.42

Pearson correlation analysis showed that trait positive empathy was positively correlated with career resilience (*r* = 0.654, *p* < 0.001) and professional identity (*r* = 0.486, *p* < 0.001). In addition, career resilience was positively correlated with professional identity (*r* = 0.557, *p* < 0.001). Detailed correlation coefficients are presented in Table 3.

**Table 3:** Pearson correlations among trait positive empathy, career resilience, and professional identity.

Variables	Trait positive empathy	Career resilience	Professional identity
Trait positive empathy	1		
Career resilience	0.654^***^	1	
Professional identity	0.486^***^	0.557^***^	1

***p < 0.001.

**Figure 3:**
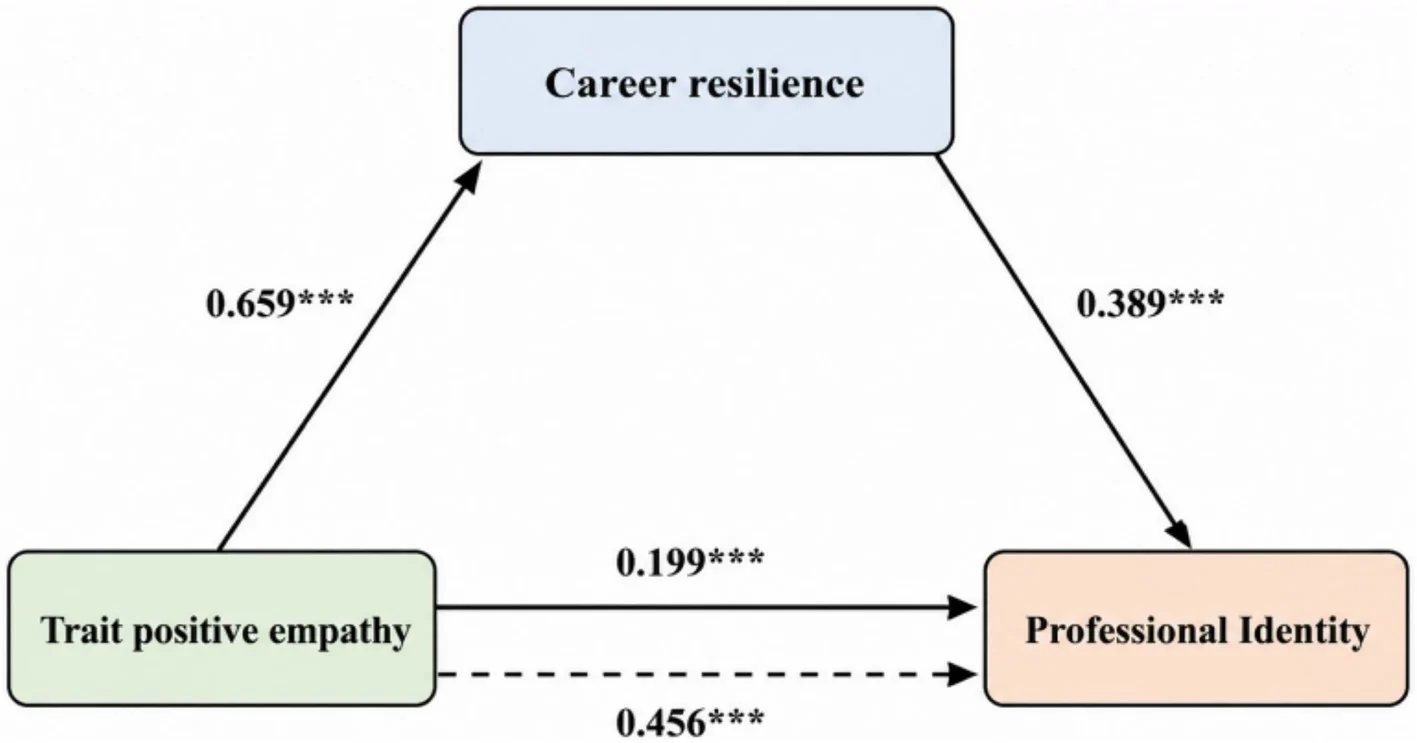
Mediating role of career resilience in the relationship between trait positive empathy and professional identity. ***p < 0.001.

Mediation analysis showed that trait positive empathy was significantly positively associated with career resilience (*β* = 0.659, *p* < 0.001), while career resilience was significantly positively associated with professional identity (*β* = 0.389, *p* < 0.001). Trait positive empathy also showed a significant positive association with professional identity (*β* = 0.199, *p* < 0.001). Bootstrap analysis further showed that the indirect association through career resilience was 0.256, with a 95% confidence interval of 0.190–0.317, which did not include zero, suggesting that the indirect association was statistically significant. The indirect association accounted for 56.23% of the total association, suggesting that career resilience partially explained the relationship between trait positive empathy and professional identity. Detailed mediation results are presented in Figure 3 and Table 4. Detailed bootstrap estimates are presented in Supplementary Table S1.

**Table 4:** Summary of the mediating role of career resilience.

Path	Direction	Interpretation
Trait positive empathy → Career resilience	Positive	Higher trait positive empathy was associated with higher career resilience.
Career resilience → Professional identity	Positive	Higher career resilience was associated with higher professional identity.
Trait positive empathy → Career resilience → Professional identity	Positive indirect association	More than half of the association between trait positive empathy and professional identity was explained through career resilience.

## Discussion

This study explored the current status of trait positive empathy among nursing undergraduates. The results showed that the total score of trait positive empathy among nursing undergraduates was 28.07 ± 4.59, with a mean item score of 4.01 ± 0.66, suggesting a moderately high level overall. This finding is consistent with the trend reported by Tunc et al. regarding empathy levels among nursing students (Ghazwani et al., 2023). This result may be related to the distribution of students across different academic years and the characteristics of various educational stages included in this study. In the present study, first- and second-year students accounted for a relatively large proportion of the sample. At this stage, students mainly engage in theoretical learning, and the curriculum includes relatively more content related to humanistic nursing, nurse–patient communication, and professional cognition. During this period, students generally maintain strong professional enthusiasm and positive career expectations, making them more likely to focus on the humanistic value of nursing and the significance of helping others. In addition, because they have not yet been extensively exposed to real clinical environments, their experiences with high-intensity workloads, complex nurse–patient relationships, and emotional labor remain relatively limited. As a result, they may be more likely to maintain sensitivity and responsiveness to patients’ positive emotions, which may be associated with higher overall levels of trait positive empathy. However, it should be noted that a moderately high level does not necessarily indicate that trait positive empathy remains stable among nursing students over time. Although no significant differences were observed across academic years in the present study, previous studies have suggested that prolonged clinical exposure may be associated with nursing students’ empathy-related characteristics. Under prolonged academic and clinical stress, nursing students may be more vulnerable to emotional exhaustion, which may be related to reduced attention and to others’ positive emotions. Therefore, nursing educators should implement targeted strategies focused on emotional support and stress-management resources throughout clinical training, to support the sustained development of trait positive empathy among nursing undergraduates.

This study explored the current status of career resilience among nursing undergraduates. The results showed that the total score of career resilience among nursing undergraduates was 3.97 ± 0.66, suggesting a moderately high level overall. This finding is consistent with the results reported by Ayed et al. regarding psychological resilience among nursing students (Ayed et al., 2025). This suggests that most nursing undergraduates possess a certain degree of psychological recovery ability and professional coping capacity when facing academic stress, clinical adaptation, and future career development pressures. The findings may be related to nursing students’ gradual exposure to real clinical nursing environments during undergraduate education. First-year students mainly focus on theoretical learning and professional cognition. At this stage, they have not yet fully entered clinical environments, and their understanding of nursing is largely derived from classroom education and professional ideals. Therefore, they are more likely to focus on the professional value and humanistic significance of nursing. While continuing theoretical learning, second- and third-year students gradually enter clinical practicum settings, where they begin to encounter real nursing situations and realize that nursing requires not only professional skills, but also the ability to cope with nurse–patient communication, teamwork, and fast-paced working environments. Fourth-year students enter long-term clinical internships and simultaneously face multiple challenges, including nursing licensure examinations, graduation, and employment preparation. These findings suggest that stress and future career-related pressures may gradually increase over time, and nursing students are exposed to the realities of the nursing profession progressively across different stages of education rather than entering high-pressure clinical environments all at once (Jia et al., 2025). During this process, some students may experience disappointment due to the discrepancy between professional ideals and clinical realities. However, others may gradually develop relatively stable psychological adjustment abilities and professional coping strategies through solving clinical problems, receiving guidance and feedback from clinical instructors, and adapting to clinical work rhythms (Tang et al., 2025). Therefore, the moderately high level of career resilience observed in this study may reflect the presence of certain adaptive psychological resources among nursing undergraduates during long-term professional learning and clinical practice. However, a moderately high level does not necessarily indicate that nursing students are fully capable of coping with all forms of clinical stress. Particularly during senior academic years, multiple stressors—including clinical internships, licensure examinations, graduation arrangements, and employment decisions—may overlap. Without adequate support, nursing students may still experience career confusion, psychological exhaustion, or even professional withdrawal tendencies. Therefore, nursing education should provide targeted support according to the characteristics and needs of students at different educational stages to support career resilience among nursing undergraduates.

This study explored the current status of professional identity among nursing undergraduates. The results showed that the total professional identity score among nursing undergraduates was 60.72 ± 10.44, with a mean item score of 3.57 ± 0.61, suggesting a moderately high overall level. This finding is consistent with the trend reported by Liu et al. regarding professional identity among nursing undergraduates (Chen et al., 2026b). Among the dimensions, “social comparison and self-reflection” and “social persuasion” showed relatively higher scores, whereas “perceived benefits of remaining versus leaving the profession” showed a relatively lower score. This suggests that nursing undergraduates generally recognize the nursing profession itself, but still exhibit some hesitation regarding long-term engagement in nursing careers. This finding may be related to the inconsistency between “recognition of professional value” and “recognition of career development” during the formation of professional identity among nursing students. Before entering nursing programs, most nursing students develop their understanding of the nursing profession mainly through family influence, social publicity, and external evaluations. Therefore, they are more likely to form positive perceptions regarding job stability and the social value of nursing (Mao et al., 2021). In addition, during undergraduate education, encouragement from educators, gratitude from patients, and positive feedback in clinical settings may be associated with stronger recognition of the value of nursing, which may explain the relatively higher scores in the dimensions of “social comparison and self-reflection” and “social persuasion.” However, with increasing clinical exposure, nursing students’ perceptions of the nursing profession may gradually shift from idealized expectations to more realistic understandings (Adamson and Dewar, 2015). During clinical practice, students become more directly exposed to realities such as heavy workloads, continuous emotional labor, and complex nurse–patient communication (Eren and DINç, 2025). At the same time, some students gradually realize that career development in nursing may also be associated with factors such as educational background, professional title promotion, hospital level, and departmental differences. Therefore, although students recognize the social value of nursing, they may develop new concerns regarding long-term career rewards and professional development opportunities, which may be related to the relatively lower score in the dimension of “perceived benefits of remaining versus leaving the profession.” In addition, some international studies have shown that nursing students’ choice of major is more often based on personal interest and autonomous decision-making (Chen et al., 2026a). In China, however, some students still choose nursing majors due to family recommendations, employment stability, or admission adjustment policies. Consequently, their professional identity may be more susceptible to fluctuations resulting from real clinical experiences (Yasin et al., 2026). These findings suggest that, in addition to strengthening professional value education, nursing education should also support students in developing a more comprehensive understanding of the nursing profession to support professional identity among nursing undergraduates.

The results of this study showed that trait positive empathy was significantly positively correlated with professional identity (*r* = 0.486, *p* < 0.01), suggesting that higher levels of trait positive empathy were associated with higher levels of professional identity among nursing undergraduates, which supports Hypothesis 1 (H1). One possible explanation for this finding is that students with higher levels of trait positive empathy are more likely to perceive positive feedback from patients, educators, and peers during clinical interactions, which may be associated with a stronger sense of the meaning and value of nursing work. Previous studies have indicated that positive feedback may be associated with individuals’ sense of professional belonging and professional engagement (Dosil-Diaz et al., 2025). Therefore, these students may be more likely to develop positive evaluations of the nursing profession and show stronger recognition of nursing as a career. The present study also demonstrated a significant positive correlation between trait positive empathy and career resilience (*r* = 0.654, *p* < 0.01), suggesting that higher levels of trait positive empathy were associated with higher levels of career resilience, which supports Hypothesis 3 (H3). During clinical learning, nursing students are continuously exposed to challenges such as academic stress, role transition, nurse–patient communication, and adaptation to clinical environments. Students with higher levels of trait positive empathy may be more likely to perceive positive feedback during clinical learning and interpersonal interactions, thereby maintaining relatively positive and stable psychological states when facing stress (Leger et al., 2020). Fredrickson’s Broaden-and-Build Theory suggests that positive emotions are associated with the accumulation of psychological resources and individuals’ ability to adapt to their environments (Andreychik and Migliaccio, 2015). Therefore, students with higher levels of trait positive empathy may possess stronger emotional regulation and stress adaptation abilities when facing clinical stress, which may be associated with higher levels of career resilience. In addition, this study found that career resilience was significantly positively correlated with professional identity (*r* = 0.557, *p* < 0.01), indicating that higher levels of career resilience were associated with higher levels of professional identity among nursing undergraduates, which supports Hypothesis 2 (H2). Previous studies have shown that individuals with higher levels of career resilience are more likely to maintain positive professional attitudes and sustained professional engagement when facing occupational stress and career setbacks (Bogerd et al., 2023). After entering clinical settings, nursing students are gradually exposed to the realities of nursing work, including heavy workloads, high levels of responsibility, and continuous emotional labor. Consequently, their professional identity may be negatively associated with clinical stress and adverse professional experiences. However, students with higher levels of career resilience are generally more likely to show positive adjustment when facing stress through proactive adjustment and positive coping strategies, thereby maintaining stronger recognition of the nursing profession and greater confidence in their future careers, which may be related to higher levels of professional identity.

The findings of this study demonstrated that career resilience played a partial mediating role in the relationship between trait positive empathy and professional identity. The indirect association accounted for 56.23% of the total association, which was higher than the direct association (43.77%), thereby supporting Hypothesis 4 (H4). These findings suggest that the association between trait positive empathy and professional identity may be partially explained by career resilience. In other words, although trait positive empathy may be associated with positive professional attitudes, this association is not entirely direct and may instead be further translated through students’ psychological adaptability during academic learning and clinical exposure. According to Fredrickson’s Broaden-and-Build Theory, positive emotions may broaden individuals’ cognitive and behavioral repertoires and be associated with the accumulation of psychological and social resources (Andreychik and Migliaccio, 2015). Nursing undergraduates with higher levels of trait positive empathy are more likely to perceive positive information such as patients’ gratitude, educators’ recognition, peer support, and perceptions of professional value, which may be associated with more positive professional experiences (Fang et al., 2026). These positive experiences may be related to lower perceived stress associated with academic learning and clinical exposure and to stronger persistence and recovery ability when facing difficulties, thereby supporting the development of career resilience. Career resilience may be further associated with nursing undergraduates’ understanding and recognition of the nursing profession. During professional learning and clinical practice, nursing students are exposed not only to the value and meaning of nursing work, but also to practical stressors such as heavy workloads, nurse–patient communication, role transition, and clinical uncertainty (Sanchis-Giménez et al., 2023). Without sufficient career resilience, students’ initially idealized perceptions of the nursing profession may be challenged. In contrast, students with higher levels of career resilience are more capable of balancing professional expectations with realistic pressures, thereby maintaining relatively stable professional attitudes and confidence in their future careers (Salameh et al., 2025). Therefore, career resilience may represent an important psychological pathway through which trait positive empathy is associated with professional identity. The findings of this study suggest that the development of professional identity may be related not only to their recognition of the value of nursing, but also to their psychological adaptability when facing occupational stress. Therefore, in addition to cultivating professional knowledge and clinical skills, nursing educators should also emphasize the development of positive psychological resources and career resilience among students. Strategies such as increasing positive clinical experiences, strengthening support from educators and clinical instructors, and providing emotional regulation training and career adaptation education may help nursing students better transition from professional cognition to professional adaptation, thereby supporting their professional identity (Wille et al., 2025).

## Conclusion

The findings of this study demonstrated that trait positive empathy was directly associated with professional identity among nursing undergraduates and was also indirectly associated with professional identity through career resilience. These findings suggest that nursing educators and clinical instructors should pay greater attention to the cultivation of positive psychological resources and career resilience among nursing students, thereby supporting their adaptability when facing clinical stress and professional challenges and further supporting the development and stability of professional identity.

## Limitations

This study employed a convenience sampling method and included only nursing undergraduates from one university in Henan Province. As a non-probability sampling approach, convenience sampling may introduce selection bias, thereby limiting the representativeness and generalizability of the findings to broader populations of nursing undergraduates. Future studies should expand the sampling scope and adopt multicenter and probability sampling methods to improve the representativeness and generalizability of the findings. In addition, this study used a cross-sectional design and collected data at a single time point; therefore, it could not dynamically reflect changes in the relationships among trait positive empathy, career resilience, and professional identity over time. Therefore, the associations and mediating role observed in this study should be interpreted within the cross-sectional statistical model rather than as evidence of temporal causality. Future longitudinal studies are warranted to further explore the dynamic relationships and potential pathways among these three variables.

## Data Availability

The raw data supporting the conclusions of this article will be made available by the authors, without undue reservation.

## References

[ref1] Adamson E. Dewar B. (2015). Compassionate care: student nurses' learning through reflection and the use of story. Nurse Educ. Pract. 15, 155–161. doi: 10.1016/j.nepr.2014.08.002, 25754833

[ref2] Althubaiti A. (2023). Sample size determination: a practical guide for health researchers. J. Gen. Fam. Med. 24, 72–78. doi: 10.1002/jgf2.600, 36909790 PMC10000262

[ref3] Andreychik M. R. Migliaccio N. (2015). Empathizing with others' pain versus empathizing with others' joy: examining the separability of positive and negative empathy and their relation to different types of social behaviors and social emotions. Basic Appl. Soc. Psychol. 37, 274–291. doi: 10.1080/01973533.2015.1071256

[ref4] Aryuwat P. Asp M. LöVENMARK A. Radabutr M. Holmgren J. (2023). An integrative review of resilience among nursing students in the context of nursing education. Nurs. Open 10, 2793–2818. doi: 10.1002/nop2.1559, 36564896 PMC10077422

[ref5] Ayed M. Ejheisheh M. A. Ayed A. Batran A. (2025). Understanding the relationship between resilience and psychological well-being among nursing students in Palestine. BMC Nurs. 24. doi: 10.1186/s12912-025-03566-z, 40634916 PMC12239407

[ref6] Bogerd R. Debets M. P. M. Keuken D. G. Hassink R. J. Henriques J. P. S. Lombarts K. M. J. M. H. (2023). The relationship between physicians' self-kindness and professional fulfillment and the mediating role of personal resilience and work-home interference: a cross-sectional study. PLoS One 18. doi: 10.1371/journal.pone.0284507, 37093877 PMC10124859

[ref7] Chen Y. P. Han X. Y. Li W. Chen Y. Han X. Hao P. (2026a). Factors influencing professional identity formation in male nursing students in China: a qualitative study from a life course perspective. Nurse Educ. Pract. 92. doi: 10.1016/j.nepr.2026.104752, 41666675

[ref8] Chen Y. P. Hao P. J. Lou Y. Chen Y. Hao P. Han X. (2026b). Exploring the influencing factors of professional identity in geriatric nursing among undergraduate nursing interns from a life course perspective: a qualitative study. J. Nurs. Manag. 2026. doi: 10.1155/jonm/5532147, 41635894 PMC12862497

[ref9] De Vries N. De Winter P. Drost-Goossens S. Vermeulen H. van Oostveen C. (2026). Key factors associated with nurse retention and how they work: a mixed-methods study. Int. J. Nurs. Stud. Adv. 10. doi: 10.1016/j.ijnsa.2026.100480, 41623357 PMC12857392

[ref10] Dosil-Diaz C. Bugallo-Carrera C. Gandoy-Crego M. Gerbaudo-González N. (2025). Attitude and personality trait changes among nursing students and professionally active nurses working with older adults. Front. Public Health 13. doi: 10.3389/fpubh.2025.1581281, 41030359 PMC12476986

[ref11] Eren N. B. Dinç L. (2025). Investigating the relationship between emotional labor levels, job satisfaction, and burnout among nurse academicians: a structural equation modeling approach. Front. Psychol. 16. doi: 10.3389/fpsyg.2025.1677531, 41415351 PMC12709624

[ref12] Ewert C. Buechner A. Schröder-Abé M. (2024). Stress perception and coping as mediators of the link between self-compassion and affective well-being? Evidence from two longitudinal studies. Mindfulness 15, 372–388. doi: 10.1007/s12671-023-02295-1

[ref13] Fang Y. P. Yu W. X. Zhao Q. G. Fang Y. Yu W. Zhao Q. . (2026). Preferences of undergraduate nursing students for value education in professional courses: a conjoint analysis. Nurse Educ. Today 158. doi: 10.1016/j.nedt.2025.106942, 41325671

[ref14] Fitzgerald A. M. Butcher D. (2025). Professional identity among soon-to-graduate nursing students: an international comparison study. J. Nurs. Educ. 64, 689–695. doi: 10.3928/01484834-20250624-04 41191418

[ref15] Ghazwani S. Alshowkan A. Alsalah N. (2023). A study of empathy levels among nursing interns: a cross-sectional study. BMC Nurs. 22. doi: 10.1186/s12912-023-01381-y, 37391749 PMC10311812

[ref16] Hao Y. F. (2011). Study of the Model of Self-Education in Enhancing the Level of Professional Identity and Professional Self-Efficacy in Nurse Students. [D]. The Second Military Medical University.

[ref17] Jia X. L. Ren S. S. Huang Y. Y. Shi P.-L. Mi Y. Zhang M. . (2025). Transition shock among nursing students during clinical practice: a scoping review protocol. BMJ Open 15. doi: 10.1136/bmjopen-2024-098112, 40132842 PMC11934382

[ref18] Leger K. A. Charles S. T. Almeida D. M. (2020). Positive emotions experienced on days of stress are associated with less same-day and next-day negative emotion. Affect. Sci. 1, 20–27. doi: 10.1007/s42761-019-00001-w, 34113848 PMC8188996

[ref19] London M. Noe R. A. (1997). London's career motivation theory: an update on measurement and research. J. Career Assess. 5, 61–80. doi: 10.1177/106907279700500105

[ref20] Malik A. Sinha N. (2026). Integrating positive psychology into nursing education: a strengths-based intervention. J. Appl. Res. High. Educ. 18, 1238–1258. doi: 10.1108/jarhe-12-2024-0738

[ref21] Mao A. M. Lu S. E. Lin Y. Mao A. He M. (2021). A scoping review on the influencing factors and development process of professional identity among nursing students and nurses. J. Prof. Nurs. 37, 391–398. doi: 10.1016/j.profnurs.2020.04.018, 33867096

[ref22] Morelli S. A. Lieberman M. D. Zaki J. (2015). The emerging study of positive empathy. Soc. Personal. Psychol. Compass 9, 57–68. doi: 10.1111/spc3.12157

[ref23] Nie S. X. Sun C. Wang L. Nie S. Wang X. (2021). The professional identity of nursing students and their intention to leave the nursing profession during the coronavirus disease (COVID-19) pandemic. J. Nurs. Res. 29:e139. doi: 10.1097/jnr.0000000000000424, 33534354

[ref24] Pan J. D. Ho K. Y. Liu H. L. Huang J.-Y. Zhang X.-L. Zeng Q.-M. . (2024). Implementation and effectiveness of a nurse navigation programme based on noddings' care theory in first-year undergraduate nursing students for professional identity and career planning: a quasi-experimental study. Nurse Educ. Pract. 75. doi: 10.1016/j.nepr.2024.103900 38277802

[ref25] Roth L. H. O. Bencker C. Lorenz J. Laireiter A.-R. (2024). Testing the validity of the broaden-and build theory of positive emotions: a network analytic approach. Front. Psychol. 15. doi: 10.3389/fpsyg.2024.1405272, 39380763 PMC11458511

[ref26] Salameh B. Abdallah J. Malak M. Z. Shehadeh A. Hammad B. (2025). Alarm fatigue and its association with perceived stress, resilience, and coping behaviors among Palestinian nursing students during clinical internship in critical care units: a cross-sectional study. BMC Psychol. 13:486. doi: 10.1186/s40359-025-02809-7, 40341074 PMC12063433

[ref27] Sanchis-Giménez L. Lacomba-Trejo L. Prado-Gascó V. Giménez-Espert M. d. C. (2023). Attitudes towards communication in nursing students and nurses: are social skills and emotional intelligence important? Healthcare 11. doi: 10.3390/healthcare11081119, 37107953 PMC10137617

[ref28] Su J. J. Paguio J. T. Masika G. M. Wang M. Redding S. R. (2021). Learning compassionate care: experiences of nursing students. Nurse Educ. Pract. 53. doi: 10.1016/j.nepr.2021.103092, 34049091

[ref29] Tang Y. Y. Chen X. W. Liao Y. Y. Tang Y. Chen X. Liao Y. . (2025). Status and associations of transition shock among nursing students during clinical practice: a cross-sectional study. PLoS One 20. doi: 10.1371/journal.pone.0313524, 39903753 PMC11793765

[ref30] Telle N. T. Pfister H. R. (2016). Positive empathy and prosocial behavior: a neglected link. Emotion Rev. 8, 154–163. doi: 10.1177/1754073915586817

[ref31] VON Elm E. Altman D. G. Egger M. Pocock S. J. Gøtzsche P. C. Vandenbroucke J. P. (2008). The strengthening the reporting of observational studies in epidemiology (STROBE) statement: guidelines for reporting observational studies. J. Clin. Epidemiol. 61, 344–349. doi: 10.1016/j.jclinepi.2007.11.008, 18313558

[ref32] Wille E. Opheim H. M. S. Princeton D. M. Kisa S. Hjerpaasen K. J. (2025). Building resilience and competence in bachelor nursing students: a narrative review based on social cognitive theory. Nurs. Rep. 15. doi: 10.3390/nursrep15070253, 40710947 PMC12298541

[ref33] Wu X. N. Lu Y. Z. Zeng Y. H. Wu X. Lu Y. Zeng Y. . (2024). Personality portraits, resilience, and professional identity among nursing students: a cross-sectional study. BMC Nurs. 23. doi: 10.1186/s12912-024-02007-7, 38907353 PMC11191206

[ref34] Wu X. A. Lu Y. Z. Zhang Q. S. Wu X. Lu Y. Zhang Q. . (2022). Stress/resource complex, sense of coherence and professional identity among nursing students: a latent profile and mediation analysis. Psychol. Res. Behav. Manag. 15, 2409–2420. doi: 10.2147/PRBM.S378088, 36065461 PMC9440726

[ref35] Wu Y. Yu W. Z. Wu X. Y. Yu W. Wu X. Wan H. . (2020). Psychological resilience and positive coping styles among Chinese undergraduate students: a cross-sectional study. BMC Psychol. 8. doi: 10.1186/s40359-020-00444-y, 32762769 PMC7406959

[ref36] Yasin Y. M. Al-Hamad A. Metersky K. Durepos P. (2026). Factors influencing nursing career choice and stress among generation Z nursing students in New Brunswick: a cross-sectional study. Can. J. Nurs. Res. doi: 10.1177/08445621261436549, 41919626 PMC13454403

[ref37] Yi T. Dai S. M. Tao J. R. Dai S. Tao J. Chen Y. . (2025). Profiles of academic resilience and associated factors among undergraduate nursing students: a latent profile analysis. J. Prof. Nurs. 61, 116–126. doi: 10.1016/j.profnurs.2025.09.014, 41318170

[ref38] Zhang J. J. Zhao C. J. Li F. Y. Zhang J. Zhao C. Li F. . (2023). Longitudinal relationships among career adaptability, resilience, and career commitment in Chinese nursing undergraduates: testing differences in career interest between cross-lagged models. BMC Nurs. 22. doi: 10.1186/s12912-023-01224-w, 36964586 PMC10036963

[ref39] Zhao S. Liang Q. L. Tao H. Liang Q. Fan S. Xia Y. . (2024). Transition shock among nursing interns and its relationship with patient safety attitudes, professional identity and climate of caring: a cross-sectional study. BMC Nurs. 23. doi: 10.1186/s12912-024-01722-5, 38267964 PMC10807204

[ref40] Zhong Y. Ma H. Zhang C. C. Jiang Q.-Y. Li J. Liao C.-J. . (2024). Professional identity, job satisfaction, and turnover intention among Chinese novice nurses: a cross-sectional study. Medicine 103. doi: 10.1097/MD.0000000000036903, 38241583 PMC10798701

