# Supplementary material for: Trait positive empathy and professional identity among nursing undergraduates: the mediating role of career resilience
Source: Front Psychol. 2026 Aug 10;17:1894726. doi: 10.3389/fpsyg.2026.1894726 (PMC13500270; doi:10.3389/fpsyg.2026.1894726)
Supplement: Supplementary file 1 [file Table_1.docx]

Supplementary Table S1. Detailed Bootstrap Results of the Indirect Association

| **Association type** | **Path** | **Association Value（B）** | **Boot SE** | **95% CI** | | **Proportion**  **(%)** |
| --- | --- | --- | --- | --- | --- | --- |
|  |  |  |  | **LLCI** | **ULCI** |  |
| Indirect association | TPE→CR→PI | 0.256 | 0.031 | 0.190 | 0.317 | 56.23 |
| Direct association | TPE→PI | 0.199 | 0.041 | 0.120 | 0.279 | 43.77 |
| Total association | TPE→PI | 0.456 | 0.033 | 0.391 | 0.520 | 100.00 |

Note. TPE = trait positive empathy; CR = career resilience; PI = professional identity; LLCI = lower limit confidence interval; ULCI = upper limit confidence interval.
****p*＜0．001
